# A cardiopulmonary bypass with deep hypothermic circulatory arrest rat model for the investigation of the systemic inflammation response and induced organ damage

**DOI:** 10.1186/s12950-014-0026-3

**Published:** 2014-08-12

**Authors:** Melanie Engels, Esra Bilgic, Antonio Pinto, Edwin Vasquez, Lena Wollschläger, Holger Steinbrenner, Kristine Kellermann, Payam Akhyari, Artur Lichtenberg, Udo Boeken

**Affiliations:** 1 Department of Cardiovascular Surgery, Faculty of Medicine, Heinrich Heine University, Moorenstr. 5, Duesseldorf, D – 40225, Germany; 2 Institute for Biochemistry and Molecular Biology I, Heinrich Heine University, Duesseldorf, Germany; 3 Clinic for Anaesthesiology, Klinikum rechts der Isar, Technische Universität, Munich, Germany

**Keywords:** SIRS, Ischaemia/reperfusion, Cardiopulmonary bypass, Deep hypothermic circulatory arrest, STAT3, IL-6

## Abstract

**Background:**

Cardiopulmonary bypass (CPB) is a commonly used technique in cardiac surgery. CPB is however associated with a strong induction of systemic inflammatory response syndrome (SIRS) which in conjunction with ischemia and reperfusion may lead to multiple organ failure.

The aim of the study was to establish and characterize a CPB rat model incorporating deep hypothermic circulatory arrest with a specific focus on the extent of the inflammatory reactions and organ damage as a groundwork for novel therapeutics against SIRS and I/R induced organ injury.

**Materials and methods:**

Male Wistar rats (n = 6) were cannulated for CPB, connected to a heart-lung-machine (HLM) and cooled to a temperature of 16°C before they underwent 45 minutes of deep hypothermic circulatory arrest with global ischaemia. Arrest was followed by rewarming and 60 minutes of reperfusion. Haemodynamic and vital parameters were recorded throughout the CPB procedure. Only animals displaying sinus rhythm throughout reperfusion were utilized for analysis. Rats were euthanized and tissue samples were harvested. Blood gas analysis was performed and blood samples were taken. Induction of organ damage was examined by analysis of protein levels and phosphorylation status of kinases and stress proteins. Results were compared to animals (n = 6) which did not undergo CPB.

**Results:**

CPB induced leucocytosis and an increase of interleukin-6 and TNF-α plasma values indicating an inflammatory response. Markers of tissue damage and dysfunction, such as troponin T, creatinine and AST were elevated. Phosphorylation of STAT3 was induced in all examined organs. Activation of MAPK and induction of heat shock proteins occurred in an organ-specific manner with most pronounced effects in heart, lungs and kidneys.

**Conclusions:**

The presented CPB rat model shows the induction of SIRS and activation of specific signalling cascades. SIRS seems not to be provoked during DHCA and is elicited mainly during reperfusion. This model might be suitable to test the efficacy of therapeutics applied in major heart surgery with and without DHCA.

## Background

Cardiopulmonary bypass (CPB) is a required technique for major cardiovascular surgery. As a core component of “on pump” surgery, extracorporeal circulation and oxygenation of blood is applied. Both processes take place in a heart lung machine (HLM). The exposure of the blood to artificial surfaces activates a variety of signalling cascades which induce an inflammatory response, first described as whole body inflammation syndrome [1]. Evolutionary needed for wound healing, under unfavourable haemodynamic conditions as it can occur during CPB, this may lead in 2-10% of all cases to a systemic inflammatory response syndrome (SIRS), which may further aggravate to multiple organ dysfunction syndrome (MODS) [2],[3]. SIRS is mediated primarily by the cells of the innate immune system. Later anti-inflammatory compensatory effects are promoted by the adaptive immune response [4]. The “one hit model” proposes that a severe SIRS alone is able to induce MODS [5]. Induction of leucocytosis and secretion of the cytokines TNF-α and IL-1β by activated monocytes and macrophages are the first signs for SIRS followed by a raise in IL-6 plasma level and a switch in T_h1_/T_h2_ cell balance [4],[6]. The activation of the immune system is at least partially responsible for collateral tissue damage observed after CPB [7], but it has to be unlinked from the pure ischemia/reperfusion process. Ischaemia/reperfusion injuries are caused to major tissues, primarily cardiovascular and visceral organs and the central nervous system [8]. Those injuries are mediated by Ca^2+^-overload and reactive oxygen species (ROS), which amongst others are generated by infiltrating macrophages [9],[10] and mainly contribute to morbidity and mortality after successful surgery [11]. The extent of I/R-induced tissue damage is not only restricted to the cardiovascular system but also affects the kidneys, the respiratory system, the liver, the central nervous system and the intestine [12]. Until now, treatment of I/R damage on clinical scale is limited to an increase of fibrinolysis which might indirectly decrease the postoperative inflammatory response [13], whereas therapies that directly suppress I/R damage are lacking. One approach would be to counteract the induction of SIRS following the “one hit model”. For this purpose, we established a rat model which relies on preceding experiments of Jungwirth et al. [14].

Following the van’t Hoff equation, lowering the temperature by 10°C decreases the metabolic rate of the myocardium by 50%. In accordance with this concept known since the 19^th^ century, hypothermia was successfully introduced into cardiac surgery for myocardial protection by Lewis and Taufic in 1953 [15]. Deep hypothermic circulatory Arrest (DHCA) has proven to be an effective mean of ischemia protection not only for the cardiovascular system but even more for the cerebrospinal and renal system [16].

Extending the aforementioned models, we elucidated biochemical events leading to the systemic inflammatory response associated with CPB and DHCA in multiple organs in a clinically relevant approach. We hypothesized that SIRS is not induced by DHCA but it is mainly affected by the following reperfusion, in which organ damage becomes apparent. The here presented model enabled us to determine common hemodynamic parameters and to assess a variety of circulating surrogate markers for the inflammatory response [17] as well as early alterations in protein levels and/or phosphorylation of MAPKs (Mitogen activated protein kinase), STAT3 (Signal transducer and activator of transcription) and Heat-Shock-Proteins, e.g. heme oxygenase 1 (HO-1) and heat-shock-protein 70 (HSP70), on the organ level. Elevated biosynthesis and/or activation of these proteins are triggered by I/R-induced inflammatory signals in the heart and other organs. They mediate key signalling events following I/R and the extent of their induction/activation determines the outcome of tissue adaption and inflammation after CPB and DHCA [10],[18],[19]. MAPK, STAT3, HO-1 and HSP70 are mediators of the I/R- and cytokine-induced organ damage and also potential targets for selective inhibitors or activators which may supress SIRS [2],[19]-[21]. Therefore we considered it as our primary goal to determine the organ-specific signalling status in target organs possibly affected by MODS. Based on information on hemodynamic and metabolic parameters combined with molecular I/R-induced alterations in various organs, the presented rat model appears to be a suitable experimental platform for the in-depth investigation of SIRS and associated signalling events. This may contribute to improve the outcome of patients undergoing CPB and DHCA in cardiac surgery.

## Methods

All reagents had analytical grade purity and were acquired from Sigma-Aldrich if not stated otherwise.

### Animals

This study was approved by the local authority LANUV (Landesamt für Natur, Umwelt und Verbraucherschutz NRW) and carried out in accordance with the German guidelines of laboratory animal care.

All experiments were performed with male Wistar rats weighing between 500 and 600 g, which were purchased from Janvier Breeding Center (Le Genest St. Isle, France). They were housed at the Institute of Animal Experiments of the Heinrich-Heine-University in stables with a temperature of 22°C, a relative humidity of 55% and a day/night cycle of 12/12 hours, with food and water ad libitum. Rats were randomly divided into two groups. The first group was subjected to an operative procedure and exposed to I/R (I/R; n = 6), whereas the second group consisted of healthy animals that were not exposed to I/R (H; n = 6). Healthy animals were not cannulated, but directly transcardially perfused to guarantee best organ preservation for western blot analysis.

### Ischaemia/reperfusion model

This model was established by Jungwirth et al. and adopted for our project with modifications as described below. Rats of the I/R-group were treated as follows: After anaesthetisation in an exsiccator rats were endotracheally intubated with a 14G cannula (Vasofix Safety, Braun, Melsungen, Germany) and mechanically ventilated (70 strokes/min., 45% O_2_/balance N_2_, PaCO_2_ of 35-45 mm Hg). During subsequent surgical preparation anaesthesia was maintained with 2.0-3.0 vol % isoflurane. Monitoring was maintained using a rectal temperature sensor, an oxygen saturation-clip at the right hindpaw and continuous electrocardiogram (ECG). The median sacral artery was cannulated with a 20G cannula (Vasofix Safety, Braun, Melsungen, Germany), which served as the arterial inflow cannula for the CPB circuit. Systemic administration of 200 IU heparin and 0.5 μg of fentanyl followed the placement of the catheter. Mean arterial blood pressure (MAP) was monitored via cannulation of the femoral artery. A 4.5 multi-orifice cannula was pleaded into the right atrium through the right external jugular vein and served as the venous outflow. The custom made heart lung machine circuit consisted of a venous reservoir, a roller pump and an oxygenator, which was built of two plexiglas plates surrounding a disposable three layer hollow fiber membrane with a gas exchange area of 558 cm^2^ (M. Humbs, Valley, Germany). A scheme of the CPB circuit is shown in Additional file 1: Figure S1 of the supplementary data. The CPB circuit was primed with 15 ml of 6% hydroxyethyl starch (HAES 6%, KabiPac, Fresenius Kabi GmbH, Bad Homburg, Germany). Through the venous catheter blood of the jugular vein flew into the venous reservoir leading the blood through the peristaltic pump into the membrane oxygenator where the gas exchange occurred. From there on the enriched blood returned to the animal via the arterial inflow cannula.

To secure a uniform time frame for the cannulation in all experiments, 90 minutes after placing the arterial catheter the rats were connected to the HLM, and CPB was induced. The flow rate started with 160 to 180/kg^−1^/min^−1^ (which is similar to the physiologic cardiac output of the rat) and was gradually decreased or increased by half during the cooling and rewarming period, respectively. During the CPB fentanyl (0.5 μl/h) and cisartracurium (Nimbex® 0.8 mg/h, GlaxoSmithKline GmbH & Co. KG, München, Germany) were administered over the venous reservoir and the rats were ventilated with 10 strokes/min. To secure the perfusion of the organs the MAP was maintained above 40 mmHg via small doses of norepinephrinhydrochloride (0.02 mg/ml Arterenol®, Sanofi-Aventis, Frankfurt, Germany), if necessary. Moreover, CO_2_, bicarbonate (NaBiC 8.4%, Braun, Melsungen, Germany) or trometamol (Tris 36.34%, Braun, Melsungen, Germany) were administered to adjust for pH fluctuations (α-stat blood gas regime), if required. No blood transfusions were given.

**Figure 1:**
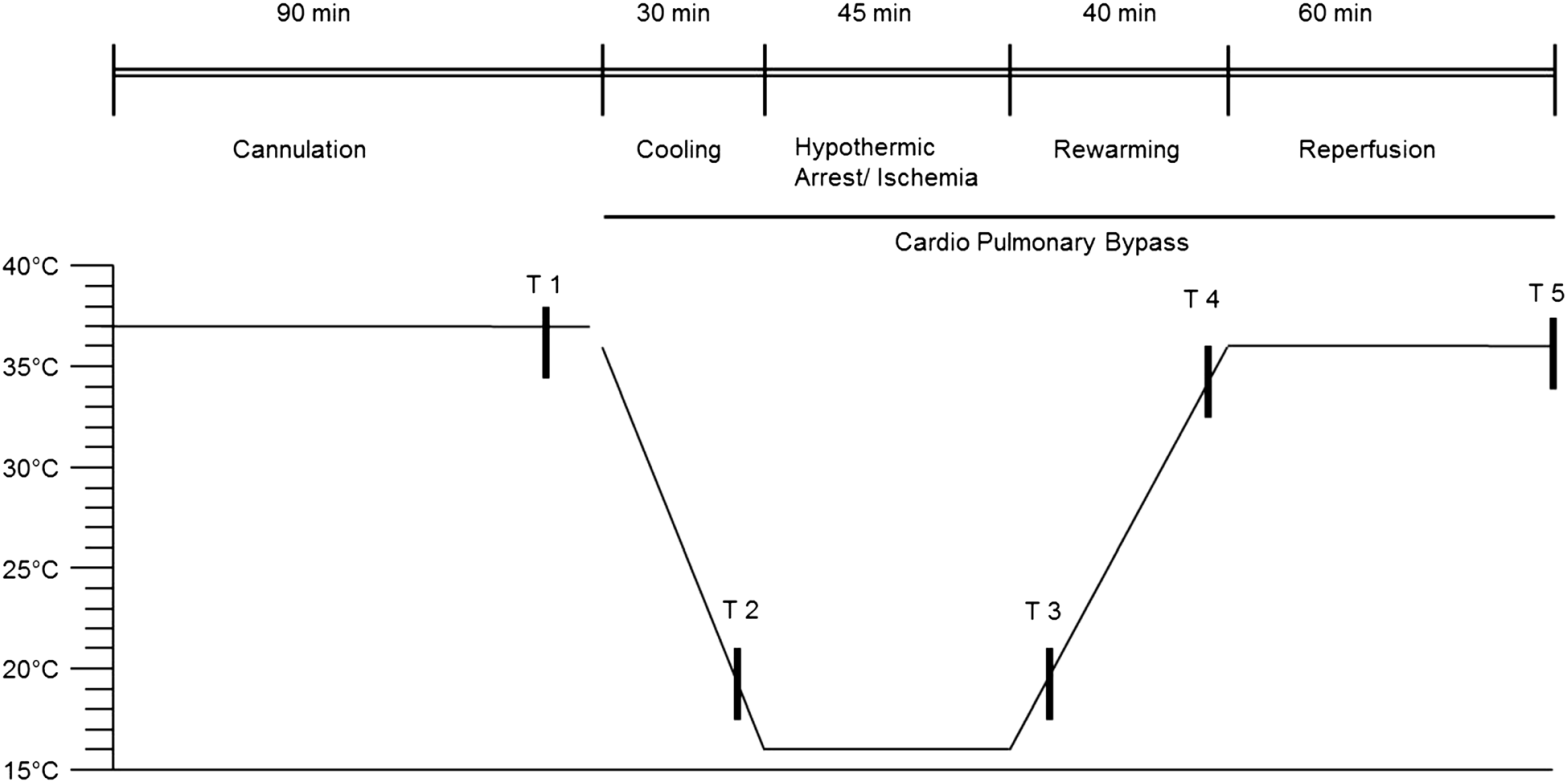
**Schematic illustration of the experimental time and temperature flow.** T1-T5 represent predefined time points for blood sampling. T1 = before the start of CPB; T2 = after 25 minutes of cooling; T3 = at 20°C of rewarming; T4 = at 35°C of rewarming; T5 = after 60 minutes of reperfusion.

Systemic cooling was carried out by a heat exchanger and additional ice bags were used for topical cooling to achieve a rectal temperature of 16°C within 30 minutes. At a rectal temperature of 16°C CPB, anaesthesia and ventilation were interrupted and the rats were exposed to 45 minutes of DHCA to expose all organs to ischaemia. After 45 minutes of DHCA the CPB was re-started and the rats were slowly rewarmed to a rectal temperature of at least 35.5°C within 40 minutes. An infrared lamp was employed additionally, if required. By reaching 35.5°C the rats were re-perfused for further 60 minutes before CPB was terminated and organ harvesting took place. A schematic illustration of the experimental time and temperature flow is shown in Figure 1.

### Harvesting of samples

Before the start of CPB (T1), after 25 minutes of cooling (T2), during rewarming at a rectal temperature of 20°C (T3) and 35°C (T4) and after 60 minutes of reperfusion (T5) arterial blood gas analyses were performed and the vital signs were documented. Additionally, blood samples were taken at T1, T2 and T5. They were transferred to heparin-containing tubes and kept at room temperature for 30 minutes to allow coagulation before centrifuging at 4000g. Plasma aliquots were snap-frozen and stored at -80°C.

For harvesting of organs, animals were perfused with NaCl and organs were removed in the following order: heart, lung, liver, and kidney. All organs were immediately snap-frozen in liquid nitrogen for subsequent molecular analysis.

In order to illustrate the study design and the time points taken for data collection throughout the experiment, the experimental time and temperature flow is given in a scheme (Figure 1). The experimental setup was designed to mimic standard procedures in the clinical scenario of cardiothoracic surgery using CPB and DHCA. Similarly, time points of blood sampling have been set to meet critical transition points during CPB. After an initial period of establishment, six animals of the I/R group were evaluated.

Healthy animals (H) were anaesthetised by injection of pentobarbital. Blood samples were taken by puncture of the left ventricle after anaesthetisation and the rats were perfused with NaCl for 3-5 minutes until organs could be harvested. Animals in the H group did not undergo any further surgical treatment.

### Analysis of metabolic parameters in plasma samples

Using blood plasma samples taken before CPB (T1), after 25 minutes of cooling (T2) and after 60 minutes of reperfusion (T5) following parameters were determined by the Central Institute of Clinical Chemistry and Laboratory Medicine of the University Hospital Duesseldorf: lactate, urea, aspartate transaminase (AST), alanine transaminase (ALT), lactate dehydrogenase (LDH), creatinine and potassium. These parameters were measured spectrophotometrically using commercially available standard Roche-Hitachi methodology (Roche Diagnostics GmbH, Mannheim, Germany). Plasma interleukin-6 (IL-6) and TNF-α levels were determined using an ELISA (Gen-Probe DIACLONE SAS, France) according to the manufacturer’s instructions. High sensitive troponin, c-reactive protein (CRP), creatine kinase (CK) and MB isoform of CK (CK-MB) were determined in plasma samples by a partner laboratory specialised in clinical diagnostics (Laboklin; Bad Kissingen, Germany) using ELISAs according to the manufacturer’s instructions.

### Analysis of molecular parameters in tissue samples

Immunoblot analysis of proteins in tissue samples was performed as previously described [22]. Briefly, a portion of each tissue was lysed in M-Per Mammalian Protein Extraction Reagent (Thermo Scientific Pierce, Rockford, IL) containing protease inhibitors (Merck, Darmstadt, Germany) and phosphatase inhibitors (Sigma-Aldrich, Taufkirchen, Germany). The protein content of the lysates was measured by DC Protein assay (Bio-Rad, München, Germany) with bovine serum albumin as standard. Lysates were boiled in Laemmli-loading-buffer and loaded either onto 10% or 14% SDS-PAGE gels. After electrophoresis the gels were transferred to PVDF-membranes (Hybond-P, General Electrics Healthcare, München, Germany). Equal loading was controlled by Ponceau S staining. After blocking the membranes with 5% non-fat dry milk for 60 minutes, the following primary antibodies were applied: anti-phospho-ERK1/2, anti-total-ERK1/2, anti-phospho-p38 MAPK, anti-total-p38 MAPK, anti-phospho- JNK, anti-total-JNK, anti-phospho-STAT3, anti-total-STAT3, pan-Cadherin (all obtained from Cell Signaling Technology, Beverly, MA). Moreover, anti-HSP 70 (StressMarq Biosciences Inc., Victoria, Canada), anti-HO-1 (Epitomics, Burlingame, California), and anti-β-actin (Sigma-Aldrich, Taufkirchen, Germany) were used. As secondary antibodies, HRP-coupled anti-rabbit IgG (Dianova, Hamburg, Germany) and anti-mouse IgG (Thermo Scientific Pierce, Rockford, IL) antibodies were used. As chemoluminiscence reagents Supersignal Pico and Femto (Thermo Scientific Pierce, Rockford, IL) were used. Signals were detected on x-ray films (GE healthcare, Pittsburgh, PA).

### Statistical analysis

One-way Anova for repeated measurement was used to analyse changes at different time points (T1, T2 and T5) followed by a post hoc Tukey test. Nonparametrical analysis by Friedman Test gave similar results. Analysis between healthy animals and T1 of the I/R group was done by Student’s t-Test. All analyses were performed by Graphpad Prism 5.0.

## Results

### Haemodynamic parameters

Table 1 displays the haemodynamic and physiological parameters of the animals in the I/R group. CPB priming with 15 ml 6% hydroxyethyl starch resulted in an expected decrease of haemoglobin concentration from 12.3 (±0.4) g/dl before CPB to 4.5 (±1.0) g/dl at the end of the entire experiment and a decrease of the haematocrit from 35.8 (±1.9) % before CPB to 9.4 (±2.6) % at the end of the experiment.

**Table 1:** Haemodynamic and physiological parameters

	**T1**	**T2**	**T3**	**T4**	**T5**
**Haematocrit (%)**	35.8 (±1.9)	17.3 (±1.0)	14.3 (±2.4)	11.9 (±3.4)	9.4 (±2.6)
**Haemoglobin (g/dl)**	12.3 (±0.4)	6.4 (±0.3)	5.9 (±0.5)	5.7 (±0.6)	4.5 (±1.0)
**WBC (x10**^**3**^**/mm**^**3**^**)**	5.0 (±2.1)	1.9 (±0.7)	1.7 (±0.7)	3.1 (±0.7)	2.7 (±0.9)
**WBC/Hct (x10**^**3**^**/mm**^**3**^**)**	5.0 (±2.1)	3.7 (±1.3)	3.8 (±1.5)	11.9 (±3.9)	24.2 (±14.4)
**WBC/Hct ratio**	1.0 (±0.0)	1.2 (±0.5)	1.0 (±0.3)	8.1 (±3.1)	11.9 (±7.2)
**Heart rate (bpm)**	294.7 (±4.9)	44.7 (±10.2)	82.5 (±27.7)	208.0 (±26.7)	228.3 (±52.2)
**MAP (mmHg)**	60.5 (±5.7)	53.7 (±7.4)	64.8 (±9.4)	51.8 (±4.9)	59.7 (±3.5)
**sO**_**2**_**(%)**	95.8 (±0.7)	95.3 (±3.8)	96.0 (±0.0)	100.0 (±0.0)	87.5 (±6.5)
**Temperature (°C)**	36.1 (±0.3)	17.5 (±0.7)	20.0 (±0.0)	35.0 (±0.0)	36.4 (±0.4)
**pO**_**2**_**(mmHg)**	48.2 (±0.9)	47.7 (±3.3)	47.3 (±5.2)	48.8 (±4.4)	44.4 (±4.8)
**pCO**_**2**_**(mmHg)**	2.7 (±0.4)	0.3 (±0.0)	0.5 (±0.2)	1.0 (±0.2)	1.1 (±0.3)
**pH**	7.27 (±0.03)	7.09 (±0.19)	7.39 (±0.21)	7.33 (±0.10)	7.35 (±0.07)
**BE (mmol/L)**	−5.6 (±1.12)	−7.7 (±3.4)	−12.7 (±2.4)	−12.5 (±3.2)	−10.0 (±3.1)
**HCO**_**3**_**(mmol/L)**	20.7 (±1.2)	21.8 (±0.9)	12.5 (±1.5)	13.8 (±3.4)	15.2 (±3.0)

At defined time points before CPB (T1), after 25 minutes of cooling (T2), at 20°C during the rewarming (T3), at 35°C during the rewarming (T4) and after 60 minutes of reperfusion (T5) haemodynamic and physiological parameters are measured. WBC = white blood cells; Hct = haematocrit; WBC/Hct ratio = WBC count as a value normalized to the respective Hct and normalized to the start values; bpm = beats per minute; MAP = mean arterial pressure; sO2 = arterial oxygen saturation; pO2 = partial pressure of oxygen; pCO2 = partial pressure of carbon dioxide; BE = base excess; HCO3 = standard bicarbonate. Data represent means ± SEM of six independent experiments.

Furthermore, a leucocytosis during the rewarming and reperfusion period was observed. Considering the haemodilution by the CPB priming, the leucocyte numbers were calculated in relation to the haematocrit to obtain comparable values. As the reference range of the leucocytes varies from 3 to 15 × 10^3^/mm^3^, for each animal the leucocyte count was normalised to the individual start value.

Regarding the MAP, no significant differences were observed between the different time points throughout the operation. Heart rate and temperature changes were in accordance with the gradual alternation of the flow rate during the cooling and rewarming period. Blood pH values and partial pressures remained stable or were corrected.

### Clinical biochemistry

**Figure 2:**
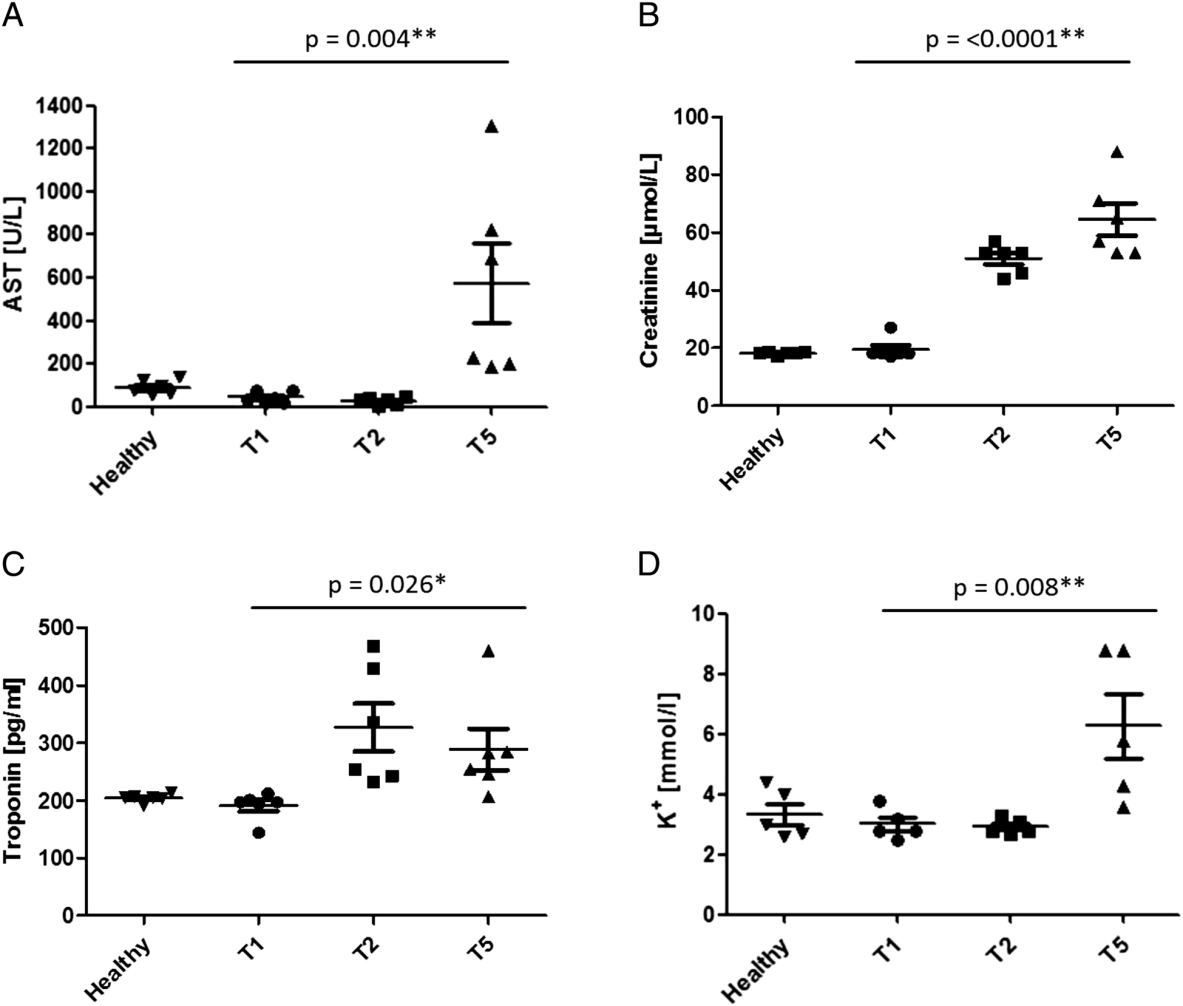
**Changes in selected clinical biochemistry parameters.** AST **(A)**, creatinine **(B)**, troponin **(C)** and potassium **(D)** were measured in plasma of healthy rats and of I/R animals before CPB (T1), after 25 minutes of cooling (T2) and after 60 minutes of reperfusion (T5). AST activity is given in units per liter (U/l). Creatinine concentration is presented in mg/dl. Troponin is given in pg/ml. Potassium concentration is presented in mg/dl. Data are the means ± SEM of six independent experiments. Significant differences by one-way ANOVA with Tukey-test post analysis is marked by one asterisk (*) for p < 0.05 and by two asterisks (**) for p < 0.01. For inter-group differences see Table 2.

The plasma samples of the healthy animals and of the time points T1, T2 and T5 were analysed for crucial clinical blood parameters as summarized in Table 2. Plasma AST, creatinine, troponin and potassium levels are exemplarily shown in Figure 2.

**Table 2:** Clinical plasma parameters at defined timepoints

	**Healthy Animals**	**T1**	**T2**	**T5**	**Healthy-T1**	**T1-T5**
**Lactate (mmol/l)**	6.22 (±1.22)	2.422 (±0.20)	5.83 (±1.04)	36.4 (±10.53)	0.03*	0.004**$#
**Troponin (pg/ml)**	204.32 (±1.22)	191.31 (±9.75)	327.02 (±41.7)	286.34 (±36.1)	0.228	0.026*§
**CK-MB (ng/ml)**	< 0.1	0.50 (±0.28)	0.34 (±0.24)	0.84 (±0.69)	n.a.	0.733
**ALT (U/l)**	43.00 (±3.73)	26.25 (±5.33)	14.6 (±0.5.76)	433.5 (±252.3)	0.03*	0.101
**AST (U/l)**	90.00 (±14.16)	43.2 (±10.2)	28.2 (±7.14)	573.5 (±184.8)	0.023*	0.004**$#
**Urea (mmol/L)**	4.42 (±0.10)	4.8 (±0.21)	5.38 (±0.92)	6.38 (±1)	0.127	0.390
**LDH (U/l)**	287.50 (±88.56)	134.5 (±100.47)	154.50 (±56.6)	3154 (±1328.98)	0.240	0.080
**Creatinine (μmol/L)**	18.0 (±0.22)	19.33 (±1.54)	51.00 (±2.02)	64.50 (±13.53)	0.412	<0.0001**§$#
**CRP (mg/ml)**	0.44 (±0.13)	0.46 (±0.08)	0.14 (±0.03)	0.17 (±0.07)	0.922	0.006**§$
**Potassium (mmol/l)**	3.03 (±0.47)	3.02 (±0.22)	2.94 (±0.11)	6.26 (±1.10)	0.980	0.008**$#
**Calcium (mmol/l)**	1.81 (±0.03)	1.69 (±0.09)	1.62 (±0.06)	1.77 (±0.21)	0.199	0.682

Values were measured in plasma of healthy animals and of I/R animals before CPB (T1), after 25 minutes of cooling (T2) and after 60 minutes of reperfusion (T5). CK = Creatine kinase; CK-MB = MB isoform of CK; AST = aspartate transaminase; ALT = alanine transaminase; LDH = lactate dehydrogenase; CRP = c-reactive protein; n.a. = not applicable. Data are the means ± SEM of six independent experiments. Significant differences were measured by ANOVA with Tukey test multiple comparison analysis. Overall significance is marked by one asterisk (*) if p < 0.05 and by two asterisks (**) if p < 0.01. Intra-group significance is marked as ^§^(T1 vs. T2), ^$^(T1 vs. T5) and ^#^(T2 vs. T5).

AST activity in plasma was decreased in I/R animals after cooling but significantly increased after reperfusion (Figure 2A) as compared to healthy animals and T1. Plasma ALT activity showed similar tendencies but these changes did not reach a statistical significance despite a clear trend (Table 2). In addition, a strong increase in Plasma LDH activity was observed after reperfusion. Compared to healthy animals and to T1 creatinine was significantly increased both, after cooling and reperfusion but remained within the reference range (Figure 2B). Urea was also increased after the cooling and reperfusion, even though it exceeded the reference range only slightly (Table 2). Levels of high sensitive troponin were explicitly increased after 25 minutes of cooling as compared to healthy animals and T1 (Figure 2C). Plasma concentrations of most electrolytes did not change during I/R with the exception of potassium that decreased after 25 minutes of cooling whereas it increased significantly after 60 minutes of reperfusion (Figure 2D). CRP levels were constant between healthy animals and T1. During CPB however, CRP levels decreased significantly at T2 and T5, possibly due to the initial priming of the system with HAES. CK-MB levels were decreased after cooling but increased after reperfusion if compared to levels of healthy animals and T1 (Table 2). Plasma lactate levels showed a slight increase after cooling but an explicit increase after 60 minutes of reperfusion as shown in Table 2. Other clinical biochemistry parameters (sodium, chloride, magnesium, creatine kinase in plasma of I/R animals) are listed in Additional file 2: Table S1 of the supplementary data.

### Increase in IL-6 and TNF-α plasma levels after reperfusion

Increased levels of the pro-inflammatory cytokines TNF-α and IL-6 can be observed during CPB.

**Figure 3:**
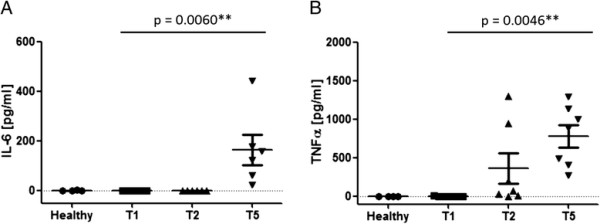
**Plasma IL-6 and TNF-α Levels.** IL-6 **(A)** and TNF-α **(B)** were measured in healthy animals and in I/R animals before CPB (T1), after 35 minutes of cooling (T2) and after 60 minutes of reperfusion (T3). Values are given as pg/ml. Data are presented as mean ± SEM of six animals. Significance was determined by one way ANOVA with Tukey-test post analysis. **A**: T5-values were significantly higher than T2-, T1- and basal animal-values; **B**: T5-values were significantly higher than T1- and basal animal-values.

IL-6 increase is associated with reperfusion and induces a variety of downstream events, e.g. cardioprotection by JAK/STAT signalling during CPB. We therefore determined the plasma IL-6 and TNF-α levels at T1, T2 and T5. Rewarming and reperfusion (T5) following DHCA led to a dramatic increase of IL-6 in all animals, causing significantly elevated values as compared to time points prior to DHCA or as compared to values observed in healthy animals (Figure 3A). Noteworthy, IL-6-levels of the T1 and T2 samples all lay under the detection level (according to the manufacturer at 19 pg/ml). TNF-α-levels were also significantly elevated after reperfusion as compared to prior time points and to healthy animals. In contrast to the IL-6-levels, TNF-α-levels were already elevated after 25 minutes of cooling (Figure 3B). Therewith the present study could demonstrate that I/R injury as applied in the presented model leads to an increase of the pro-inflammatory cytokines IL-6 and TNF-α.

### I/R-induced alterations in expression and phosphorylation status of intracellular signal mediators and heat shock proteins

Key intracellular players of the I/R related signal transduction were evaluated to further explore the validity of the presented model as a tool for scientific work on I/R. I/R modulates the kinases ERK1/2, p38 and JNK by altering their site-specific phosphorylation. Consequently, we analysed changes in phosphorylation of ERK1/2 at Y202/T204, of p38 at T180/182 and of JNK at T183/Y185 after hypothermic global ischaemia and normothermic reperfusion. Furthermore, the expression of the heat shock proteins HSP-70 and HO-1, which are induced immediately after ischaemia as organ protective mechanisms, was analysed. As a mediator of cellular inflammatory response, phosphorylation of the transcription factor STAT3 at Y705, which among others is induced by IL-6, was assessed [21].

**Figure 4:**
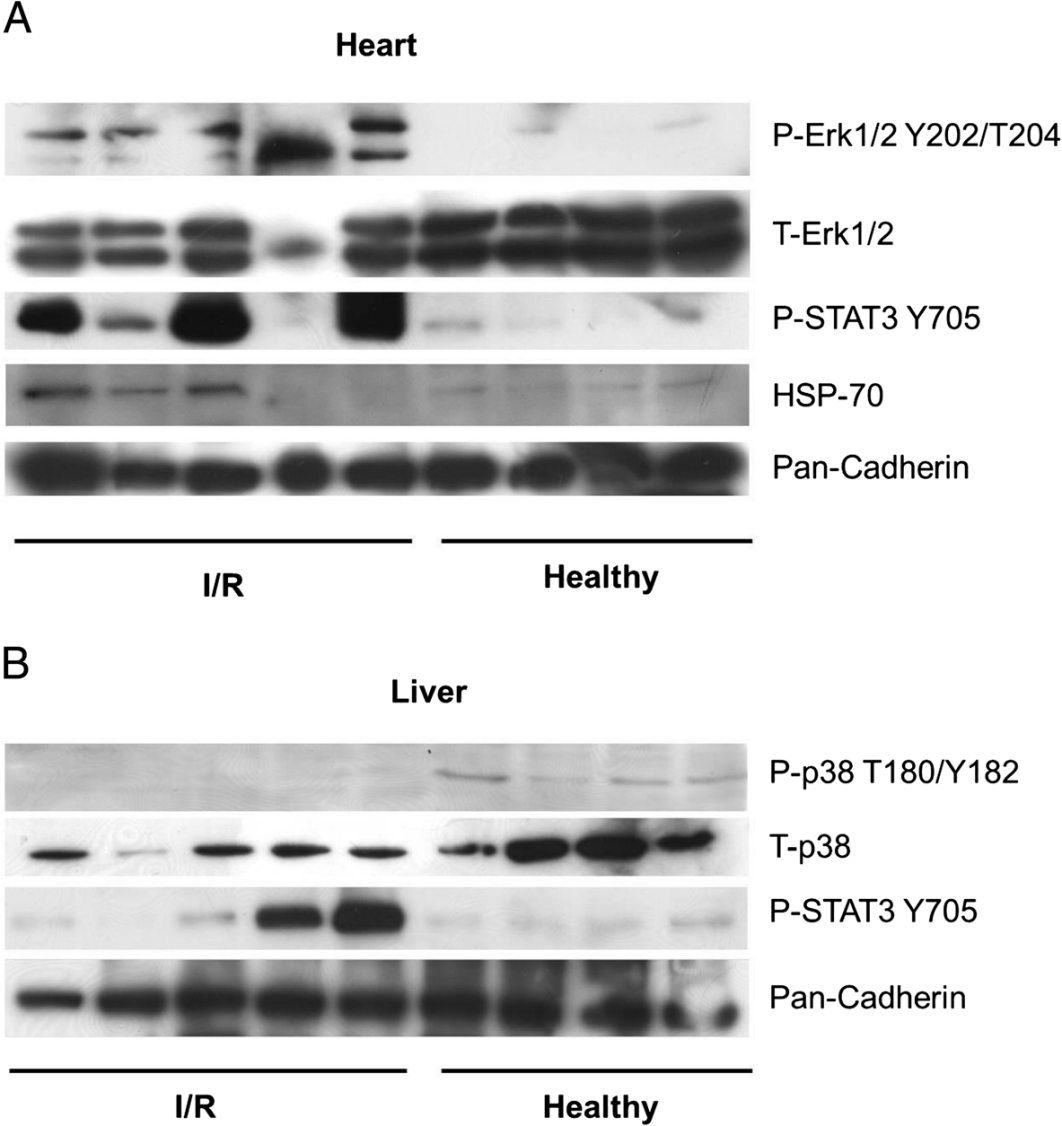
**Selected representative immunoblots of heart (A) and liver (B).** Results of protein expression analysis by western blot in heart **(A)** and liver **(B)** tissue of animals undergoing global hypothermic ischemia and reperfusion (I/R) as compared to healthy animals without I/R (Healthy).

We chose to analyse tissue samples from the heart, the lung, the liver and the kidney to demonstrate the systemic effect of I/R associated with the presented model. As a result of I/R, organ-specific phosphorylation and expression patterns could be detected, which were distinct for each of the investigated organs and will be discussed in the following paragraphs individually in detail. As a control for uniform loading and protein levels, pan-cadherin was used because it gave better results than β-actin and α-Tubulin. A brief summary is presented in Table 3. Representative blots for ERK1/2, HSP-70 and STAT3 are displayed in Figure 4A-B. The complete western blot results are shown in Additional file 3: Figure S2 and in Additional file 4: Figure S3 of the supplementary data.

**Table 3:** Compilation of the observed I/R-induced changes in protein expression or phosphorylation in different organs

	**Heart**	**Lung**	**Liver**	**Kidney**
**p-STAT3**	(↑)	(↑)	(↑)	(↑)
**p-Erk1/2**	↑	(↓)	(↓)	↓
**p-JNK**	(↑)	(↓)	-	(↑)
**p-p38**	(↓)	↓	↓	-
**HSP-70**	(↑)	↓	-	(↑)
**HO-1**	-	(↓)	-	↓

Protein expression in the I/R group is compared with healthy animals. P = phosphorylation; ↑ = increase; ↓ = decrease; (↑) = upward trend; (↓) = downward trend; - = no difference/not detectable.

### Heart

I/R induced a significant increase in the phosphorylation of cardiac ERK1/2 as compared to healthy animals (Figure 4A). Similar results have been reported for rat models of ischaemic preconditioning and were attributed to the translocation of the signal mediator protein kinase Cε from the cytosol to mitochondria [23]. Additionally, the involvement of cytokines in the present study is further indicated by increased STAT3 phosphorylation in 4 of 5 I/R animals in contrast to the healthy animals, where no phosphorylation was observed. However, when JNK was analysed, as a consequence of I/R no change could be detected in both, the total protein expression and the phosphorylation status. Furthermore, in three out of five I/R animals we observed a decrease of p38 MAPK phosphorylation, which may be due to the long reperfusion time. Similar effects have been previously observed in other rat models of isolated cardiac I/R [24]. Equally, three out of five I/R animals showed a considerable increase of HSP-70 protein expression (Figure 4A), matching the previously reported observations that HSP-70 expression is increased in myocardial infarction and I/R, potentially as a protective response. HO-1 protein expression did not differ between the two groups.

### Lung

As stated above, an increase of STAT3 protein phosphorylation was recognised in all analysed organs, including the lungs. Moreover, I/R induced a decrease of phosphorylated ERK1/2 and total ERK1/2 expression in comparison to healthy animals. Similarly, a decrease of both, phospho-JNK and total-JNK signals was detected. A decrease of phosphorylation was also visible on p38 MAPK. Based on existing reports I/R is expected to activate MAP-kinases. However, this type of regulation did not prove to be consistently predominant throughout all organs analysed in this study. Major reasons could be the dilution of WBC by the necessary hydroxyethyl starch during CPB as well as the time dependent decrease of phosphorylation of key regulator proteins after their initial activation [25]. An explicit decrease in HSP-70 expression was observed after I/R as compared with healthy animals. Additionally, four of five rats undergoing I/R showed a decrease of HO-1 protein expression. The dilution of alveolar white blood cells, having high content of HSP-70 and HO-1, might lead to reduced protein detection [26].

### Liver

When liver tissue was analysed, an increase of STAT3 phosphorylation in comparison to healthy animals was detected in animals prone to I/R (Figure 4B). Furthermore, similar to the results obtained for the lung a decrease of ERK1/2 MAPK phosphorylation was detected in the liver of I/R animals. JNK protein expression and phosphorylation did not differ between the two groups. The missing induction may imply that JNK does not contribute to I/R-associated injury nor to protective effects in the settings of this model, while under different conditions an increased JNK-activation is protective [27]. In our set-up I/R induced a strong decrease of the phosphorylation of hepatic p38 MAPK as compared with healthy animals (Figure 4B). No apparent differences in HSP-70 and HO-1 protein expression were observed between I/R and healthy animals.

### Kidney

In the kidneys, I/R also induced an increase of STAT3 protein expression. In four of five I/R animals the phosphorylation of ERK1/2 and p38 MAPK was decreased. However, there was no significant difference in p38 MAPK total protein expression detectable between the two groups. Concerning ERK1/2, the activation can be attributed to an activation of the STAT3 pathway. Furthermore, an increase of phosphorylation of JNK compared to healthy animals was observed. A consistent trend was observed with the protein expression of HSP-70, an accepted marker for renal I/R injury, which was demonstrated to be slightly elevated [22]. In contrast, a decrease in protein expression of HO-1 was detected which was not expected to occur after I/R. However, this finding may be attributed to the steady decline of HO-1 expression along the inflammatory response and increased heme release during CPB [28],23]. Interestingly, renal damage is not always observed in humans undergoing CPB [24]. Possibly, in our rat model renal damage was not accentuated, explaining the faint changes on phosphorylation and protein expression observed [29],[30].

## Discussion

Ischaemia/reperfusion (I/R) injury contributes to the development of SIRS which enhances morbidity and mortality after surgery requiring CPB and DHCA. The involved mechanisms and molecular pathways are not completely understood, yet. Thus, it is important to provide a suitable animal model which is capable of mimicking signalling events of I/R and inflammation in humans. Based on previously published animal models it therefore was the aim of this study to establish an appropriate animal model, giving special attention to SIRS associated with I/R in multiple organs.

The observed alterations of most of the analysed blood parameters showed, that they underlie an influence by CPB. The above mentioned increase in plasma AST activity is expected to occur after reperfusion, as it represents a marker for liver, skeletal and cardiac muscle damage. The observed decrease in AST activity during the cooling period might be due to haemodilution associated with CPB.

Although the increase of creatinine remained within the reference range, the course of this parameter indicates an impaired renal function developing throughout the experiment, possibly caused by I/R-induced renal tissue damage as reported [14].

The increased levels of cardiac troponin, CK-MB and LDH were in accordance with findings of other groups and have been reported in humans after CPB and I/R, respectively as compared to the levels before surgery [15].

The increase of IL-6 and TNF-α during reperfusion is associated with SIRS and may induce JAK/STAT signalling during CPB. The dramatic increase of IL-6 and TNF-α after the reperfusion is correlated with a strong leucocytosis. At the same time points CRP levels remained low, matching very well the conditions of a beginning SIRS for the intra-operative time frame we decided to investigate. CRP as a marker of the complement system activation is elevated only after one or two days after surgery [31],[32]. The present study could demonstrate that I/R injury as applied in the described model leads to an increase of the pro-inflammatory cytokines IL-6 and TNF-α, which can activate intracellular signalling [17]. For the interpretation of the above data it must be considered that we observed haemolysis in the reperfusion blood samples and that haemolysis can cause an increase of LDH, AST, ALT, potassium and CK-levels [16].

As a further result of SIRS and I/R organ-specific phosphorylation and expression patterns of stress proteins could be detected. As assessed by STAT3 phosphorylation, an inflammatory response was observed in all organs as expected [21]. Those findings are in agreement with the increased number of leucocytes and the higher IL-6 plasma levels in I/R-animals after reperfusion. Previous to the presented experiments and based on literature a number of I/R-induced alternations of the protein expression level and protein phosphorylation level were anticipated, particularly involving MAPK activation as well as heat shock protein induction. However, following our ‘cardiocentric’ and clinically derived approach those expected changes were not entirely confirmed by the presented experiments. The anticipated alterations were not present for all of the detected proteins in all organs. However, an organ-specific pattern of intracellular response to I/R has already been suggested, e.g. demonstrating divergent results for the heart as opposed to other organs [24],[33]. Especially JNK phosphorylation pattern were dissimilar for most organs, but contradictory results have been reported, indicating that JNK activation may differ in I/R injury [27],[34]. One of the major reasons for divergence in I/R-induced signalling events may be the extent of I/R that actually acts on the individual organs, but also the organ-inherent tolerance to transient ischemic periods. In case of the heart, the level of induced cardioplegia as applied in different models may represent an explanation for the differences between our results and those of other studies. In contrast, abnormal calcium levels can be excluded in our set-up as a trigger for kinase activation and heat shock protein induction, because no difference was noticed in calcium serum levels (see Table 2).

In the presented work we have chosen to undertake a final measurement of protein expression and phosphorylation at the end of the complete I/R procedure. Although this approach has proven valid to demonstrate various aspects of an ideal SIRS-I/R model, it yet may have led to a simplified picture of events occurring over the time period of the entire experiment. Likewise, the one-point detection of the read-out measures may have caused a systematic masking of kinase phosphorylation kinetics that are known to represent a highly time dependent transient effect [18],[24]. Furthermore, the truly SIRS-dependent molecular effects have to be dissected from other I/R variables by ongoing experiments. Thus, in following studies the influence of hypothermia, reperfusion and haemolysis on I/R and SIRS triggered signalling events shall be further analysed.

The following limitations may be applied to our study. Cardiac arrest was achieved by deep hypothermia, no cardioplegic solution was applied. This was done on purpose to exclude signalling induced by excessive application of potassium. Since the focus of the study centers on early signalling events which may protect from or induce organ damage, we did not investigate angiopathic and apoptotic changes induced by I/R. Moreover the transition from SIRS to MODS was not aim of this study. These points will be considered in ongoing studies.

## Conclusion

We established a CPB rat model that can reproduce common pathophysiological and molecular alterations that are associated with the induction of SIRS and the activation of specific signalling cascades. This standardised model may serve as a tool to evaluate the extent of the inflammatory reactions and organ damage associated with I/R and SIRS and to investigate the potential of novel therapeutics in a preclinical model. It might be suitable to test the efficacy of immunosuppressive therapeutics applied in major heart surgery using CPB with and without DHCA. The contribution of the different aspects of CPB might be investigated in detail, as the role of oxidative stress and inflammation might be further discriminated by analysing the involved molecular pathways.

## Abbreviations

ALT: Alanine transaminase

AST: Aspartate transaminase

CK: Creatine kinase

CK-MB: MB isoform of creatine kinase

CPB: Cardiopulmonary bypass

CRP: C-reactive protein

DHCA: Deep hypothermic circulatory arrest

ECG: Electrocardiogram

e.g.: Example give

Erk: Extracellular-signal-regulated kinases

HAES: Hydroxyethyl starch

HLM: Heart-lung-machine

HO: Heme oxygenase

HSP: Heat shock protein

IL: Interleukin

I/R: Ischemia/reperfusion

IU: International units

JNK: c-Jun N-terminal kinases

LDH: Lactate dehydrogenase

MAP: Mean arterial pressure

MAPK: Mitogen-activated protein kinases

MODS: Multi organ dysfunction syndrome

ROS: Reactive oxygen species

SIRS: Systemic inflammatory response syndrome

STAT3: Signal transducer and activator of transcription 3

TNF-α: Tumor-necrosis-factor-α

WBC: White blood cells

## Competing interests

The authors declare that they have no competing interests.

## Authors’ contributions

ME, EB, and LW carried out the in vivo experiments together with EV and also the biochemical analyses together with AP. ME and AP drafted the manuscript and contributed to the preparation of the figures. PA, HS, AL, EB and UB designed the concept of the study, contributed to the analysis and interpretation of the data, and reviewed as well as revised the manuscript. PA, AL and UB coordinated the in vivo experiments. AP and HS coordinated the work on the biochemical analyses. KK contributed significant intellectual input regarding concerning the establishment and subsequent improvement of the in vivo model and the involved physiology, was also involved in interpretation of the in vivo data, and critically reviewed and revised the manuscript. All authors read and approved the final manuscript.

## Additional files

## Supplementary Material

Additional file 1: Figure S1. Schematic representation of the applied CPB circuit. Click here for file

Additional file 2: Table S1. Additional clinical biochemistry parameters (sodium, chloride, magnesium and creatine kinase levels in plasma of I/R animals at times T1, T2 and T5). Click here for file

Additional file 3: Figure S2. I/R-induced changes in protein expression or phosphorylation in heart and lungs of rats, as analysed by immunoblotting. Click here for file

Additional file 4: Figure S3. I/R-induced changes in protein expression or phosphorylation in liver and kidneys of rats, as analysed by immunoblotting. Click here for file
