# Supplementary figures and images for: A cardiopulmonary bypass with deep hypothermic circulatory arrest rat model for the investigation of the systemic inflammation response and induced organ damage
Source: J Inflamm (Lond). 2014 Aug 12;11:26. doi: 10.1186/s12950-014-0026-3 (PMC4231204; doi:10.1186/s12950-014-0026-3)

Figure S1


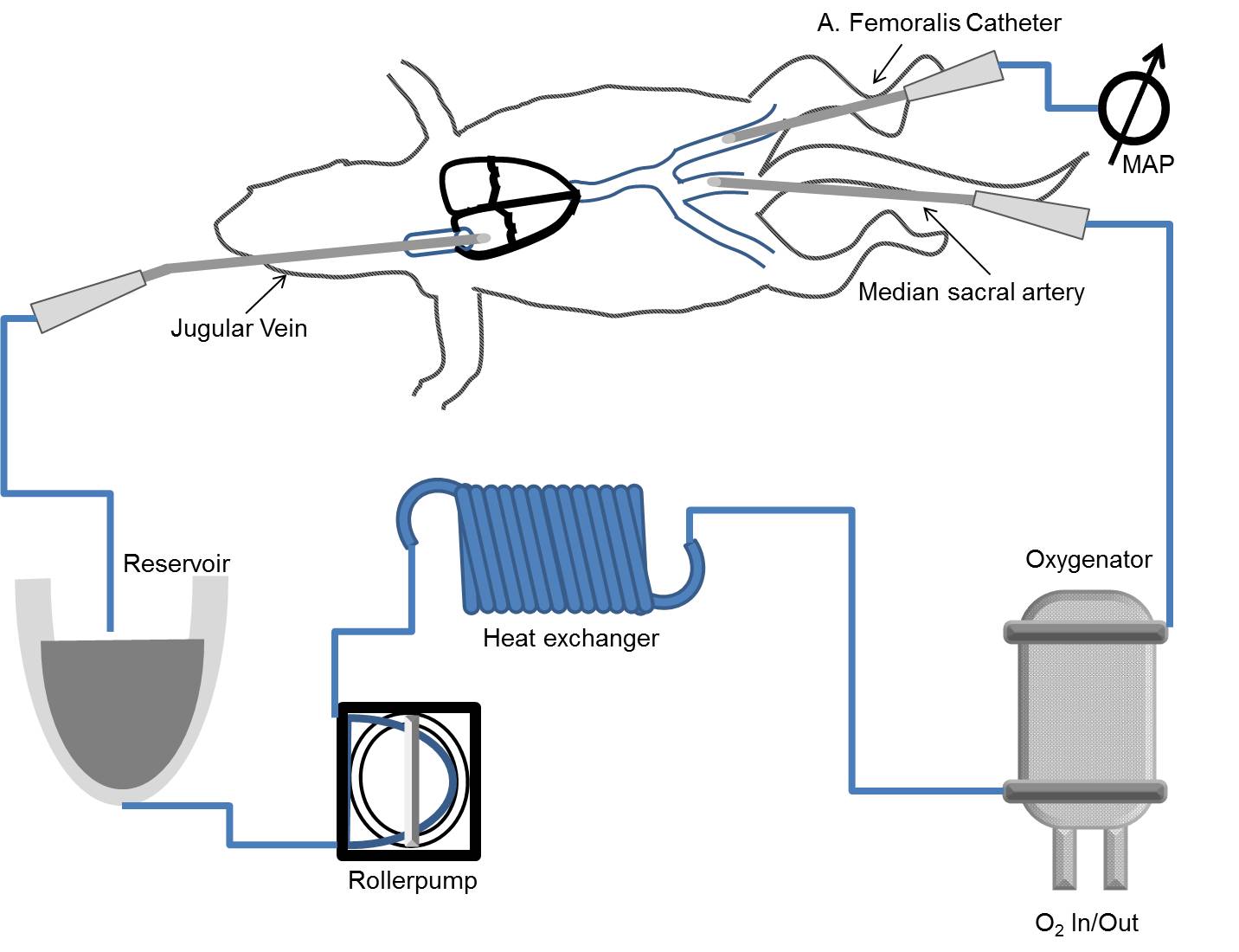

Supplement: Additional file 1: Figure S1. — Schematic representation of the applied CPB circuit. [file s12950-014-0026-3-S1.docx]

Figure S2


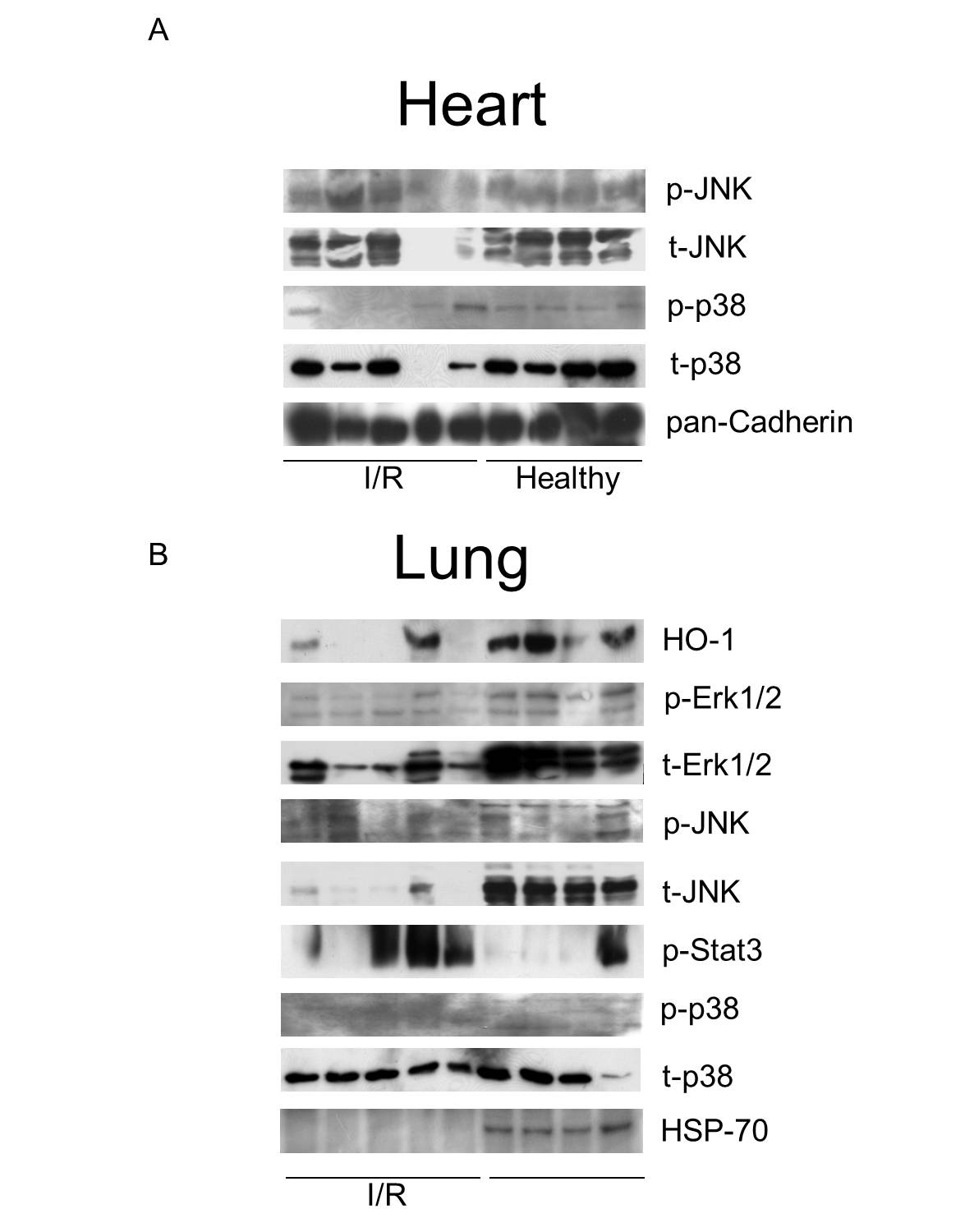

Supplement: Additional file 3: Figure S2. — I/R-induced changes in protein expression or phosphorylation in heart and lungs of rats, as analysed by immunoblotting. [file s12950-014-0026-3-S3.docx]

Figure S3


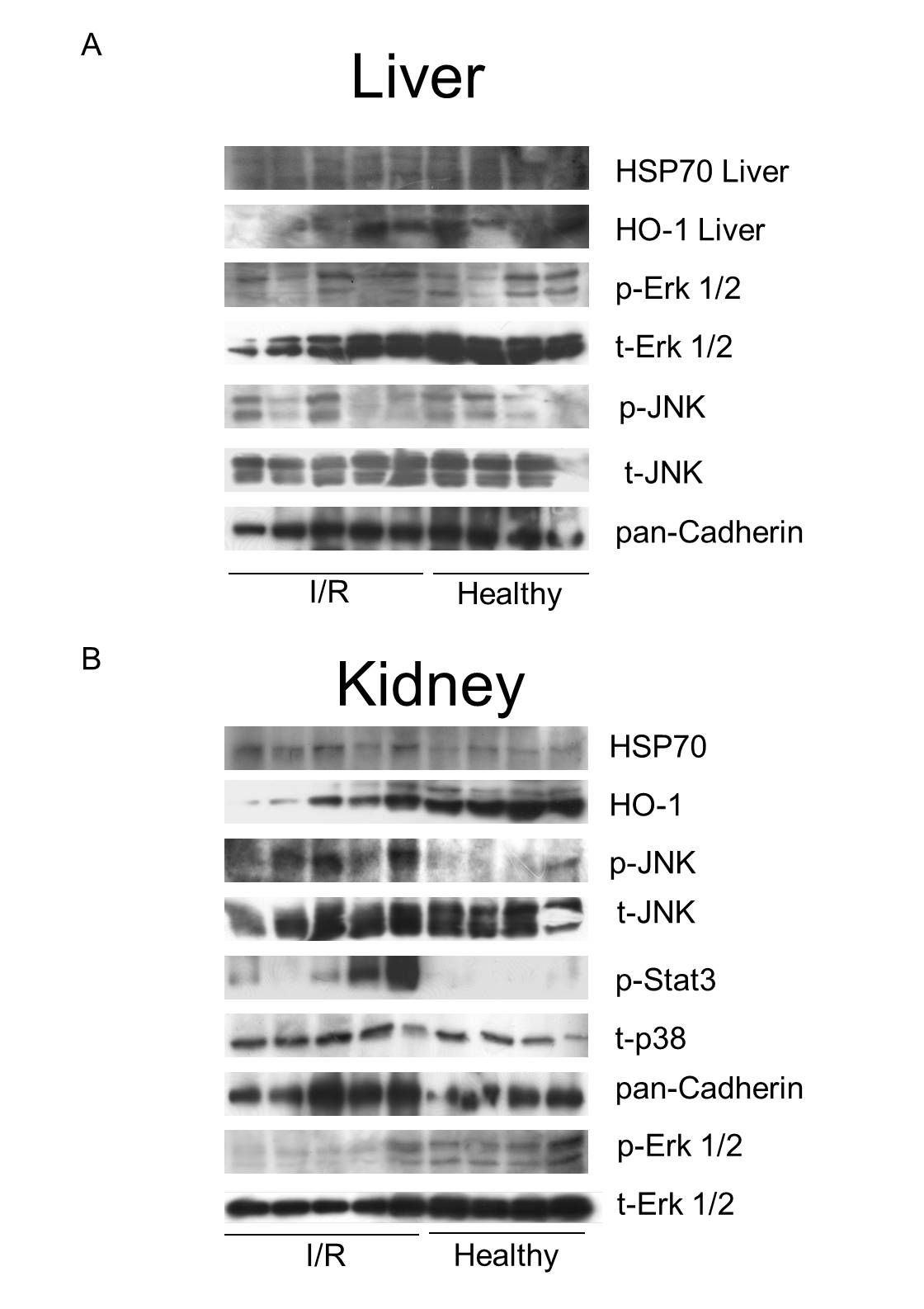

Supplement: Additional file 4: Figure S3. — I/R-induced changes in protein expression or phosphorylation in liver and kidneys of rats, as analysed by immunoblotting. [file s12950-014-0026-3-S4.docx]
