# Supplementary material for: A cardiopulmonary bypass with deep hypothermic circulatory arrest rat model for the investigation of the systemic inflammation response and induced organ damage
Source: J Inflamm (Lond). 2014 Aug 12;11:26. doi: 10.1186/s12950-014-0026-3 (PMC4231204; doi:10.1186/s12950-014-0026-3)
Supplement: Additional file 2: Table S1. — Additional clinical biochemistry parameters (sodium, chloride, magnesium and creatine kinase levels in plasma of I/R animals at times T1, T2 and T5). [file s12950-014-0026-3-S2.docx]

Supplemental data.

**Table S1 Additional clinical plasma parameters.**

|  | **T1** | **T2** | **T5** |
| --- | --- | --- | --- |
| **Sodium (mmol/l)** | 148 ( ± 0.87) | 142 ( ± 1.07) | 142 ( ± 1.90) |
| **Chloride (mmol/l)** | 87 ( ± 0.81) | 89 ( ± 2.13) | 88 ( ± 1.50) |
| **Magnesium (mmol/l)** | 1 ( ± 0.03) | 1 ( ± 0.04) | 1 ( ± 0.21) |
| **CK (U/l)** | 0 ( ± 0 .0) | 7 ( ± 1.47) | 32 ( ± 18.03) |

Values were measured before CPB (t1), after 25 minutes of cooling (T2) and after 60 minutes of reperfusion (T5). CRP = c-reactive protein; CK = creatine kinase.

**Figure S1 Schematic illustration of the rat cardiopulmonary bypass model**.

**Figure S2 Western Blots of heart and lung lysates.** Results of protein expression analysis by western blot in heart (A) and lung (B) tissue of animals undergoing global hypothermic ischemia and reperfusion (I/R) as compared to healthy animals without I/R (Healthy).

**Figure S3 Western Blots of liver and kidney lysates**. Results of protein expression analysis by western blot in liver (A) and kidney (B) tissue of animals undergoing global hypothermic ischemia and reperfusion (I/R) as compared to healthy animals without I/R (Healthy).
